# Non-Coding RNAs in Response to Drought Stress

**DOI:** 10.3390/ijms222212519

**Published:** 2021-11-20

**Authors:** Temesgen Assefa Gelaw, Neeti Sanan-Mishra

**Affiliations:** 1 Plant RNAi Biology Group, International Centre for Genetic Engineering and Biotechnology, New Delhi 110067, India; temesgen.assefa2129@gmail.com; 2 Department of Biotechnology, College of Natural and Computational Science, Debre Birhan University, Debre Birhan P.O. Box 445, Ethiopia

**Keywords:** epigenetic silencing, long non-coding RNA, miRNA, regulatory networks, stress response, water deficit

## Abstract

Drought stress causes changes in the morphological, physiological, biochemical and molecular characteristics of plants. The response to drought in different plants may vary from avoidance, tolerance and escape to recovery from stress. This response is genetically programmed and regulated in a very complex yet synchronized manner. The crucial genetic regulations mediated by non-coding RNAs (ncRNAs) have emerged as game-changers in modulating the plant responses to drought and other abiotic stresses. The ncRNAs interact with their targets to form potentially subtle regulatory networks that control multiple genes to determine the overall response of plants. Many long and small drought-responsive ncRNAs have been identified and characterized in different plant varieties. The miRNA-based research is better documented, while lncRNA and transposon-derived RNAs are relatively new, and their cellular role is beginning to be understood. In this review, we have compiled the information on the categorization of non-coding RNAs based on their biogenesis and function. We also discuss the available literature on the role of long and small non-coding RNAs in mitigating drought stress in plants.

## 1. Introduction

About 80–95% fresh biomass of non-woody plants is occupied by water, which plays an important role in many aspects of plant life. Several abiotic factors, such as low rainfall, salinity, very high or very low temperature and high intensity of light lead to water deficit in plants [1]. The reduction in water exerts stress leading to changes in the physiological, morphological, ecological, biochemical and molecular characteristics [2,3]. These changes can affect overall plant development resulting in yield reduction and/or plant loss [4]. In arid and semi-arid environments, drought is one of the most important stress factors for plants [1,4,5]. The continuous increase in environmental temperature has increased the probability of occurrence, duration and severity of drought, thereby making it challenging to meet the global food demands of the ever-increasing world population [6,7].

Drought stress tolerance is a quantitatively controlled trait in plants [8,9]. It causes changes in gene-expression patterns, water transport and osmotic balance, oxidative homeostasis and repair mechanisms. This affects the vital metabolic processes, chlorophyll synthesis and photosynthesis, decelerates seed germination, reduces stomatal movement, inhibits root development and limits nutrient uptake [3,6,10]. As sessile organisms, plants have evolved several mechanisms to withstand water stress and/or drought by inducing changes at the developmental and functional levels [5,9,11,12]. The resistance mechanisms include strategies for avoiding, escaping, tolerating and recovering from drought [13]. To tide over short periods of drought or ‘drought escape’ plants have the ability to regulate growth [10,11]. Once the stress is over, plants resume growth to overcome drought injury and this is known as drought recovery [13].

**Figure 1:**
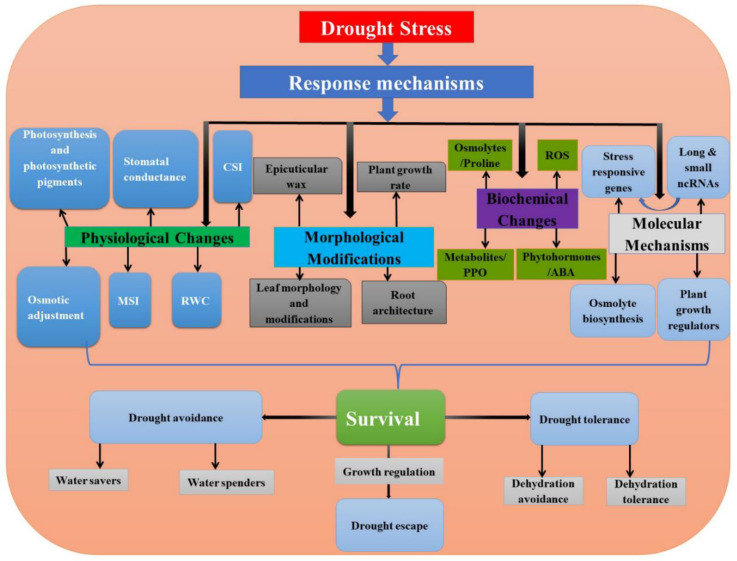
Schematic representation of different drought stress-response mechanisms operative in plants. The combined actions of these processes govern plant survival or susceptibility. The survival of plants can be grouped under drought avoidance, drought tolerance and drought escape, based on the plant response. ABA = abscisic acid, CSI = chlorophyll stability index, MSI = membrane stability index, PPO = polyphenol oxidase, ROS = reactive oxygen species, RWC = relative water content.

An important universal physiological process to overcome drought stress involves regulating stomata movement to control respiration, transpiration, photosynthesis and temperature [11,14,15]. Plants can also endure severe water-stress scenarios through osmotic adjustments and accumulation of dehydration-induced proteins [10,12,16,17]. The other changes include regulating the onset of senescence and fine-tuning of phytohormones [2,9,10,12,16] (Figure 1). Plants also modulate the redox pathway by balancing the production of antioxidant enzymes, such as including superoxide dismutase, peroxidase and ascorbate peroxidase, to scavenge the reactive oxygen species (ROS) produced during drought stress [18]. This also helps to maintain organelle stability, protect chloroplast membranes and stabilize the PSII system [19]. Therefore, it is important to identify the factors that regulate the genetic components and govern the nature of plant response.

Relatively recent studies have shown that long and small non-coding RNAs (ncRNAs) are important modulators of drought tolerance in plants [12,20,21,22,23,24]. The networking between ncRNAs and their target genes is, in turn, controlled by various other enzymatic components in the cell [9,25]. The advances in high-throughput analysis, such as RNA sequencing (RNA-Seq) and bioinformatics, have accelerated scientific research [26]. Sequence analysis has proved to be an important tool to explore the differences in response to stress between sensitive and tolerant plants, such as sorghum [27], tomato [28], coffee [29], cassava [30], peanut [31], *Populus* [32], *Trifolium* [33], wheat [34], rice [35] and maize [36,37]. This has led to the identification of stress-responsive gene expression; however, our knowledge about the regulatory processes is still limited. This review highlights important updates on the available literature on the role of long and small ncRNAs in response to drought stress response in plants.

## 2. Long Non-Coding RNAs

Over the last decade, long ncRNAs (lncRNAs), usually >200 nucleotides (nt) in size, have emerged as a pivot of genome regulation, adding a new layer of epigenetic control [38,39,40], but their clear evolutionary origins and functional specialization are still inexplicit. This group of ncRNAs lacks polypeptide-coding potential and possesses significant biochemical versatility, with each lncRNA having specific functions [39,41,42]. High-throughput RNA-Seq based investigations have primarily contributed to the identification of lncRNAs in many organisms [43]. Many investigations have been performed in humans and animals [44,45,46]; however, in plants, only a few molecules have been well characterized [39,47,48].

In eukaryotes, transcription followed by processing of the nascent RNA precedes the formation of messenger (mRNA). The biogenesis of a fully mature mRNA requires the coordinated action of enzymes that perform 7-methyl Guanosine (m7G) capping, splicing, polyadenylation, maturation, export and surveillance [49,50]. In a similar manner, primary transcripts of lncRNAs, which are produced by RNA Polymerases II or III and IV or V, undergo polyadenylation, m7G capping and splicing [24,51]. Most plant lncRNAs are polyadenylated, but in mammals and yeast, some non-polyadenylated lncRNAs have been found [52]; therefore, the presence of non-polyadenylated functional lncRNAs in plants cannot be completely ruled out [53]. The non-polyadenylated lncRNAs are processed by Ribonuclease P to generate free ends that are stabilized by the capping of small nucleolar RNA (snoRNA). In some cases, the snoRNAs have been found at both 3′ and 5′ ends. Reports have also shown that Ribonuclease P processed free ends can self-ligate to form circular structures [39,40,54,55].

Based on their biogenesis and location, the lncRNAs can be classified into several subgroups [56], as described below.

(a) Long intergenic ncRNAs (lincRNAs) are 200 to 2000 nt in length. These transcripts are derived from the intergenic region and have also been referred to as large/long intervening ncRNAs, very long intervening ncRNAs and macroRNAs [24,57]. They are polyadenylated, weakly spliced, exhibit tissue-specific expression and possess a trans-regulatory role [39,58]. These lncRNAs are characterized by rapid turnover rates, which present a challenge in understanding their functional significance [39,59]. The lincRNAs are localized at least 5 kb away from protein-coding regions and are, therefore, said to lie in gene deserts [60]. These have been sub-classified based on their association with specific regions [61,62], such as enhancer RNAs or eRNAs [52,60], upstream antisense RNA (uaRNA), promoter-associated long RNA (PALR) or promoter upstream transcripts (PROMPTs) [63] and telomeric repeat-containing RNA (TERRA). The PROMPTs and eRNAs are short-lived lncRNAs that have been identified mostly in humans.

(b) Transposable element (TE)-derived lncRNAs (TE-lncRNAs) are generated from the TEs [57]. These transcripts can sometimes act as precursors to microRNAs (miRNAs) and small interfering RNAs (siRNAs) [64,65,66]. In *Arabidopsis*, rice, maize and tomato TE-lncRNAs were reported, and their association with response to abiotic stresses was revealed [67,68,69,70]. TEs, also known as jumping genes, were first discovered in maize in the early 1950s [71]. They have the ability to copy/cut and paste themselves into other genome regions [72]. Based on the mechanism of transposition, they have been classified as Class I or RNA mediated/retro-elements and Class II or DNA elements. Class II classical TEs transpose via a cut-and-paste mechanism, while Class I TEs transpose through a reverse-transcription-based copy-and-paste mechanism. The DNA copy thus generated can get integrated anywhere in the plant genome [65,72,73]. In the maize genome, more than 85% of DNA is derived from TEs [72,74], so, correspondingly, a large number of TE-lncRNAs have been predicted to be present in maize [75,76].

(c) Intron-derived lncRNAs (incRNAs) originate from within the introns of protein-coding genes [77] and include totally intronic RNA (TIN) or partially intronic RNA (PIN). These transcripts therefore, are regulated by various transcription activation pathways [78]. The incRNAs may contain poly(A) modifications and are usually stable but they may not be highly conserved across different plant species [44,79]. It was reported that few miRNAs and snoRNAs originate from the intronic regions, so, initially, it was considered that the incRNAs may act as progenitors of the small ncRNAs; however, recent studies have confirmed their independent existence [80,81,82].

(d) Natural antisense transcripts (NATs) originate from coding regions (both exon and intron) in eukaryotic genomes and are amongst the widespread lncRNAs. They possess both *cis* and *trans*-action to regulate gene expression by silencing [83,84]. Cis-NATs are transcribed from the inverse strand of the target genomic locus to regulate the corresponding sense transcript [83]. Trans-NATs arise from a locus away from that of the target gene [83,85]. The binding of NATs triggers the production of specific siRNAs, which also exhibit a predominantly *trans* mode of action.

(e) Circular lncRNAs (circncRNAs) are highly conserved but low in abundance and are more stable than linear lncRNAs, as they cannot be degraded easily [86]. They were first characterized as non-polyadenylated circular RNAs in plant viroids [87]. CircncRNAs mostly arise in the nucleus from the back-splicing of exons in pre-mRNAs [39,86,88,89], while some arise in the cytoplasm. It is postulated that the failure of intronic lariat debranching during canonical splicing plays a role in the biogenesis of circncRNAs [88,90,91,92]. Most circncRNAs may consist of one or more extra exons and are categorized as extra-exon circular ncRNAs (eecircncRNAs), and others may be derived from the intron region of the parent gene and called circular intronic RNAs (circincRNAs) or intron retained circular ncRNAs; however, some arise from overlapping regions and are called exon-intron circncRNAs (eicincRNAs) [90,93]. Since circncRNAs are derived from the internal exon regions, they can affect the splicing of their linear counterparts. They have been shown to regulate cell development by acting as endogenous target mimics (eTM) of miRNAs, miRNA sponges [43,91], protein scaffolds or templates for protein translation. The circncRNAs present in exosomes were shown to regulate the proliferation of the respective cells [92].

### 2.1. Function of lncRNAs

It is clear that a large number of lncRNAs are transcribed in plant cells, but their molecular mechanism is largely unknown [94,95,96,97,98]. They mainly play a regulatory role by facilitating gene silencing to control transcriptional regulation and genome imprinting. These regulations are associated with diverse biological processes, such as root organogenesis [99], photo-morphogenesis [100], control of flowering time, reproduction, nutrient homeostasis [95] and so on [58,67,101]. Their expression levels vary significantly in different tissues and over different developmental stages. The lncRNAs also undergo dynamic regulatory adjustments during the response to abiotic stress [96,102,103,104,105,106] and pathogen invasion [101].

It has been generalized that the Pol IV transcribed lncRNAs serve as precursors for siRNAs, while Pol V transcribed lncRNAs act by modulating the chromatin framework [107]. The lncRNAs can act in *cis* or *trans* orientation, forming simple and complex networks. For instance, in *Arabidopsis*, ~1400 light-responsive NATs were identified, and they could act in both the same and opposite directions [106,108]. They may act as signal molecules guide molecules, precursors for miRNAs and siRNAs, regulators of pre-mRNA splicing and modulators of chromatin [70,107,109,110,111]. Some of the identified functions of lncRNAs are listed in Table 1.

#### 2.1.1. lncRNAs as Target Mimics

The lncRNAs can regulate transcription directly or by acting as target mimics of the small ncRNAs [57,124,125]. Some lncRNAs, such as *IPS1* (*induced by phosphate starvation*) and *ASCO* (*alternative splicing competitor*), contain sequences complementary to that of the miRNAs, so they can act as decoys or sponges or eTMs by competing for miRNA binding [59,112,125,126]. The mimic sites within the lncRNAs are non-cleavable and block the miRNA function, thus conferring translational regulation in *cis* orientation. Several of these target mimics are thought to have a role in plant growth and development [127].

The discovery of the *IPS1* gene in *Arabidopsis* introduced the concept of eTMs and unveiled the novel cellular mechanism behind the regulation of the miRNAs [128]. *IPS1* contains a region complementary to miR399, but it has a loop at the cleavage point of miR399. Thus, *IPS1*:miR399 forms a stable pair and quenches the silencing activity of the miRNA. Both *IPS1* and miR399 expression are induced upon phosphate starvation and *IPS1* expression seems to be required for fine-tuning of miR399 activity [112]. Subsequently, it was shown that *lncRNA23468* functions as a decoy for miR482b to compete with transcripts of *NBS-LRR* genes [129].

The *ASCO*-lncRNA binds to transcripts encoding nuclear alternative splicing regulators, *AtNSRa* and *AtNSRb* to regulate lateral root development [111]. In addition, *lncRNA16397* targets *slGRX22* (*glutaredoxin* gene) to induce the expression of *GRX21* and reduce ROS accumulation [130]. The dual regulators act to modulate gene expression during *Phytophthora infestans* infection in tomatoes.

Reports have also indicated that lncRNAs can be targeted and cleaved by the miRNAs [118,131,132]. A computational study by Fan et al. [59] found 466 maize lncRNAs as targets of 165 miRNAs and 86 lncRNAs as decoys for 58 miRNAs. In *Populus*, about 51 lncRNAs were reported as putative miRNA targets and 20 lncRNAs were reported as target mimics of the known miRNAs in response to drought stress [118]. In *Cleistogenes*, RNA-Seq analysis identified 52 lncRNAs as target mimics for miRNAs [97]. These analyses clearly showed that lncRNAs are associated with the miRNA nodes and supported their regulatory role in plants.

#### 2.1.2. lncRNAs in DNA Modification

Numerous reports have indicated that, in plants, the lncRNAs participate in the modification of DNA at different developmental stages [105] such as reproduction [133], embryogenesis [134] and organogenesis [92] under stress conditions. The classical example is provided by cold-induced incRNA, *COOLAIR*, which inhibits *FLC* (*Flowering Locus C*) during vernalization to regulate the flowering time in *Arabidopsis* [135]. The repression is achieved by enrichment of Polycomb repressive complex 2 (PRC2) and subsequent trimethylation of Histone H3 Lysine 27 (H3K27me3) at the *FLC* locus [136].

Epigenetic silencing via DNA methylation and histone modification is an important mechanism for regulating gene expression. It is specifically significant for controlling transposons, repetitive sequences and centromeric regions and for imprinting [48,70,137]. The lncRNAs can also guide gene silencing through siRNA-dependent DNA methylation [47]. The role of small RNA-directed DNA methylation (RdDM) and heterochromatinization has been well studied in plants [138,139,140]. The plant-specific RNA polymerases, RNA Pol IV and V play a crucial role in this process [24,51,141,142,143,144]. Briefly, Pol IV, along with the CLASSY chromatin remodeling factors (CCRFs) and homeodomain transcription factors, such as DTF1/ SHH1, transcribes transposons and repetitive sequences. The transcripts are converted to double-stranded RNAs (dsRNAs) by the action of RNA-dependent RNA polymerase-2 (RDR2) and the dsRNAs are processed into small ncRNAs, specifically siRNA duplexes by Dicer-like 3 (DCL3) enzyme [138,139]. These siRNAs are loaded in Argonaute 4 (AGO4)-containing complex to guide RdDM. In an alternate pathway, siRNAs are generated through Pol I–RDR6 transcription and are loaded into the AGO6 complex. At loci where Pol V is producing nascent transcripts, the siRNA-guided AGO4,6 complex interacts with the larger subunit of RNA Pol V, NRPE1 [145,146]. This complex is stabilized by the KTF1 (yeast transcription elongation factor, SPT5 homolog) to subsequently establish DNA methylation through domains rearranged methyltransferase 2 (DRM2) [141,146]. The methylated state of DNA is maintained through cell divisions through pathways catalyzed by methyltransferase1 (MET1) or chromomethylase3 (CMT3) [117,147].

It was shown that RdDM regulates the repetitive intergenic elements and their expression in maize. The RdDM function is supported by mediator of paramutation 1 (MOP1-1) in maize, which is an ortholog of At-RDR2 [148]. It was shown that MOP1 copies the RNA Pol IV transcript for processing into siRNAs [144]. In another study, 110 maize lincRNAs and 46 genic lncRNAs were predicted as precursors for *Mop1*-sensitive siRNAs [149]. In addition, 26 lincRNAs and 97 genic lncRNAs were predicted as precursors for shRNA, while one lincRNA and two genic lncRNAs were predicted as precursors for miRNA. RdDM is a complex pathway that has also been implicated with short-term and long-term stress memory [105], so further investigations are required to understand the role of lincRNAs in regulating RdDM functions in maize in response to drought and other abiotic stresses.

## 3. Small Non-Coding RNAs

The small ncRNAs comprise a number of categories among which the miRNAs and siRNAs constitute the major groups. They function as key regulators of transcriptional and post-transcriptional gene expression [139,150,151,152] and are therefore implicated in the control of various physiological and developmental processes in plants, such as growth, organ formation, phase transition, nutrient balance and stress response [10,22,153,154,155,156]. Several online tools and databases have been developed that have enabled the prediction, documentation and analysis of the small ncRNAs and their targets [48,57,124]. Deep sequencing and degradome analyses, coupled with advanced tools and databases, have driven the identification of various small ncRNAs in response to single or combined abiotic stresses [20,157,158,159].

### 3.1. Small Interfering RNAs

Overall, siRNAs are generally 21–24 nt in length and are produced by the sequential processing of long dsRNAs in a phased or non-overlapping manner. They may arise either from endogenous sources, such as TEs, repetitive elements and centromere, or exogenous sources, such as invading viruses or aberrant inverted repeats [139,160]. The siRNAs can target endogenous as well as exogenous sequences serving as the first line of host defense [161]. The long dsRNAs are processed by DCLs into mature siRNAs, which get associated with AGO protein to form the catalytic core of the RNA-induced silencing complex (RISC) to facilitate gene silencing [139,162]. The siRNA strand that directs the RISC complex is called the guide strand, while the other strand is known as the passenger strand. The passenger strand is excluded and undergoes degradation, while the guide strand directs RISC to its target transcript for cleavage. The identity of the guide and passenger strand is regulated on the basis of the thermodynamic stability of 5′ end [163,164]. The siRNAs also mediate transcriptional gene silencing through the RNA induced transcriptional silencing (RITS) complex [139].

The siRNAs are involved in regulating gene expression, maintaining genome stability and aiding plant defense. In *Arabidopsis*, DCL2 and DCL4 are involved in production of primary siRNAs from aberrant dsRNAs. DCL2 processes 22 nt siRNAs that contribute to the antiviral defense and plant development while DCL4 processes 21 nt siRNAs to initiate primary defense against invasion of viruses and transgenes [113,139]. The DCL3 processes 24 nt siRNAs to direct methylation of DNA sequences resulting in chromatin modification and transcriptional gene silencing [142,145]. The functions of DCL2 and DCL4 are partially redundant and they are also involved in biogenesis of secondary or transitive siRNAs. The secondary siRNAs are processed from dsRNA produced by the action of RDR6 and SGS3 on single stranded RNA templates that are primed by primary siRNAs [139].

Depending on their site of origin, the siRNAs are classified as repeat-associated siRNA (rasiRNA), trans-acting siRNA (tasiRNA), natural-antisense siRNA (nat-siRNA), heterochromatic siRNA (hc-siRNA) and vi-siRNA (viral siRNAs).

(a) Ra-siRNAs are derived from TEs and repetitive DNAs [165] and mainly function in the silencing of retrotransposons and various abiotic stress factors, including drought [166]. Studies in maize and *Arabidopsis* have indicated complex feedback regulatory loops between rasiRNA and their target RNAs [166,167].

(b) TasiRNAs are derived by phased cleavage of dsRNA, which is produced after miRNA-mediated cleavage of the *TAS* gene-derived transcripts. In *Arabidopsis, TAS1* and *TAS2* transcripts are targeted by miR173, *TAS3* transcripts are recognized by miR390 and *TAS4* is targeted by miR828 [168]. They play a crucial regulatory role in development through post-transcriptional silencing [169,170]. *TAS1*, *TAS2* and *TAS3* were downregulated in response to drought and salinity stress [171]. The tasiRNA-ARF (auxin response factor) module is involved in regulating flower morphogenesis under drought and salt stress [172]. In *Sorghum bicolor*, two *TAS3* gene homologs were identified to regulate the response to drought stress [170].

(c) Nat-siRNAs are a class of functional siRNAs, which originate from within the annealed regions of the natural antisense transcript (NAT) pairs [173]. Scientific evidence has indicated that *NATs* and Nat-siRNAs are involved in regulating various biological processes of plants and animals, such as phosphate homeostasis [174], stress response [175,176], chromatin remodeling and RNA editing [176,177,178,179].

(d) Hc-siRNAs are derived from heterochromatic intergenic regions including repeats and transposons [180]. The hc-siRNAs recognize the nascent Pol V-dependent transcript via base-pair complementarity and guide the DNA methylation and histone modification machinery to the loci for transcriptional gene silencing [180,181,182]. Their role has been reported in plants in response to several biotic [183,184,185,186] and abiotic stress factors [187,188].

(e) Vi-siRNAs are derived from dsRNA replicative intermediates of viruses to induce specific antiviral immunity [189]. They are generally processed from the sense strand of the viral genome [190]. Most of the vi-siRNAs have 5′ monophosphate, which indicates that vi-siRNAs can be produced by the viral RDR [191]. The role of vi-siRNAs has been reported in response to viral pathogen response in different plants, such as *Arabidopsis* [192], tomato [193], soybean [194], tobacco [195] and so on.

### 3.2. MicroRNAs

**Figure 2:**
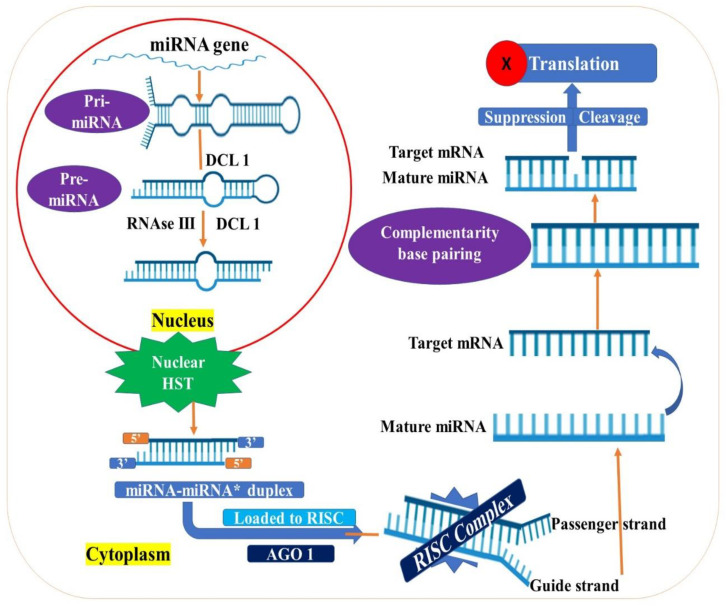
Generalized scheme to illustrate the various steps involved in plant miRNA biogenesis and activity. The miRNA genes are transcribed to primary miRNA transcripts (pri-miRNAs) of 100–120 nt long by RNA polymerase II that are then capped and polyadenylated. In the first maturation step of miRNA synthesis, pri-miRNA is cleaved by *DCL*1 in collaborative action of other enzymes to form precursor miRNA (pre-miRNA) of approximately 70–100 nt long. In the second maturation step, the hairpin structured pre-miRNA is processed by the same enzymes to mature miRNA duplex. The *DCL*1 cleavage results in the formation of a miRNA–miRNA* duplex of 21 to 24 nt containing two nucleotide 3′ overhangs and 5′ monophosphate regions. This duplex is transported out of the nucleus and into the cytoplasm, where it associates with an AGO (Argonaute) containing protein complex to form RNA induced silencing complex (RISC). The RISC is guided to the target sequence of single-stranded mRNA. Depending upon the nature of miRNA and AGO, the target mRNA is either cleaved or subjected to suppression of translation.

The miRNAs are processed from long primary transcripts that are transcribed from the genome. The steps in their biogenesis are complex and intricately regulated, as it involves the coordination of several proteins [196]. The primary transcripts (pri-miRNA) are sequentially processed by the *DCL*1 containing microprocessor complex into precursor miRNAs (pre-miRNA) and then into mature miRNAs. The steps in miRNA biogenesis are illustrated in Figure 2. Several other proteins, such as *HYL* and *SE*, are required for accurate *DCL*1 function [197]. The mature miRNA duplex is then methylated at the ends by HEN1 and transported to the cytoplasm, where it gets associated with the *AGO* containing *RISC* to form a functional complex, which can bring about transcript cleavage or suppress translation [198,199,200].

The miRNAs regulate various aspects of plant growth and development (Table 2) by regulating tissue or organ differentiation and development, shoot branching, root branching, lateral root development, panicle formation, flower development, seed development, primordial development, apical dominance, etc. [150,156,199,200,201,202,203,204,205,206]. The miRNAs also play an important role in promoting adaptation and tolerance to fluctuations in environmental conditions [207,208,209,210]. Moreover, miRNAs act in a coordinated manner by controlling the network of key genes, transcription factors and phytohormones [208,211,212,213,214].

It was seen that mutants in the miRNA biogenesis pathway exhibited an impaired response to abscisic acid (ABA), auxin and cytokinins [213,215], thus indicating the overlapping of regulatory hubs in plants. Later, it was shown that miR159 and miR164 modulated the levels of gibberellic acid (GA) and auxin, respectively [216,217,218]. The transcripts for auxin receptors, *TIR1* (transport inhibitor response-1) and *F-box protein* *2* are targeted by miR393 [219,220,221,222]. The miRNA expression levels are also modulated by hormones, as exemplified by the downregulation of miR167 after treatment with ARF [223].

The first direct evidence that miRNAs are involved in plant stress responses came from the work of Jones-Rhoades and Bartel in the year 2004. Abiotic stress-regulated miRNAs were first reported in the model plant *Arabidopsis thaliana* [224] and, by now, the stress-responsive miRNAs have been reported in almost all plant species [225,226,227,228,229]. Functional studies have also supported this role for miRNAs. For example, overexpression of miR393 reduced plant growth in drought stress by downregulating the auxin signals [230]. There are also reports on the functional involvement of the miRNA passenger strand (miRNA*) in various responses. For example, miR169g* and miR172b* were downregulated in tomato leaves under varying phosphate deficient conditions [199,231].

Plant miRNA activity is precisely controlled by the regulation of expression of miRNA genes, processing of mature miRNAs and function of miRNAs. The first level of control involves the development and tissue-specific regulation of pri-miRNA transcription in response to hormonal and environmental cues by a variety of transcription factors [232,233], such as ARF, LFY, MYC2, etc. The transcripts of many of these transcription factors are regulated by the miRNAs, indicating the existence of complex cellular feedback loops [234].

The second level of control is achieved by regulating the processing or biogenesis of mature miRNAs. This is indicated by differences in the levels of pri/pre-miRNA and mature miRNAs and by the presence of *DCL*3 dependent 24 nt long miRNAs [169,235]. The regulation of *DCL1* transcripts by miR162 and *AGO*1 transcripts by miR168 also adds to the spatial or temporal differences in miRNA activities [236]. In 2008, it was reported that overexpression of SINEs (short interspaced elements) resulted in phenotypes similar to that of miRNA-deficient mutants. Later, it was discovered that stem-loops of SINEs mimic the pre-miRNAs to bind and quench *HYL*1 [237].

The third level of control can be achieved by sorting miRNAs in different *AGO* complexes. According to the most popular hypothesis, the 5′-terminal nucleotide of miRNA guide strand determines the selection of the specific *AGO* containing *RISC* and, hence, the subsequent mode of action [238,239]. For instance, Uridine at the 5′ end supports preferential sorting with *AGO*1, whereas adenosine at the 5′ end favors sorting with *AGO*2 and *AGO*4 [240].

**Table 2:** List of conserved miRNAs and their key target genes that function in plant growth and development.

miRNAs	Target Gene	Functions	References
miR156/157	*SPL*	Phase transition from vegetative to reproductive phase; flowering	[203]
miR159	*MYB family*	Development of male reproductive organs	[216]
miR160	*ARF10, ARF16*	Controls root development and gravitropism	[213]
miR165/166	*HD-ZIPIII*	Leaf development and polarity; lamina expansion	[202]
miR166	*RDD1*	Grain size and weight	[204]
miR167	*ARF10, ARF16, ARF17*	Floral patterning; controls anther and ovule development	[241]
	*ARF6, ARF8*	Stamen and gynoecium and maturation; seed development	[214]
miR168	*AGOs*	Leaf polarity	[200]
miR169	*NF-YA*	Floral organ identity	[242]
miR172	*AP2*	Floral patterning and floral organ development; regulates the inner whorl organ differentiation	[243]
miR319	*TCP*	Leaf morphogenesis	[226]
miR390	*ARF2, ARF3, ARF4*	Leaf development, adaxial identity of leaf blade, lateral organ development and leaf senescence	[172]
miR394	*Leaf Curling Responsiveness (LCR)*	Regulation of leaf curling, shoot meristem differentiation and maintenance in abscisic acid–dependent manner	[244]
miR396	*Growth Regulating Factors (GRFs)*	Adaxial–abaxial polarity of leaf and cell proliferation	[245]
miR399	*PHO2*	Control of flowering time	[227]
miR408	*Plantacyanin*	Root development	[246]
miR444	*MADS box*	Floral patterning and development control	[247]
miR824	*MADS-box gene*	Formation of stomatal complexes in meristems	[248]
miR824	*AGL16*	Stomatal development	
miR848	*IAA28*	Root development and lateral root development	[249]
miR1218	*NAC3*	Organ separation	[250]

## 4. Role of Long and Small Non-Coding RNAs during Drought Stress

The regulatory functions of plant lncRNAs and miRNAs in plant stress response have been comprehensively studied [67,106,109,251,252]. These two classes of ncRNAs also participate in response to water deficit and drought through complex cellular pathways involving chromatin modulation, target mimicry, transcriptional regulation, hormonal signaling and by directly regulating drought-responsive genes [57,117,158,230,253,254].

### 4.1. lncRNAs in Drought Stress

Genome-wide transcriptome studies have identified several drought-responsive lncRNAs in different plant species [96,97,115,255,256,257,258]. For example, studies on the identification of drought-responsive lncRNAs in grass families detected 664 potential candidates in maize [102], 98 in rice [119], 19 in foxtail millet [259] and 1597 in switchgrass [117]. The various reports on the identification of drought-responsive lncRNAs are presented in Table 3. Most lncRNAs regulate the drought response by acting on genes participating in ethylene and ABA synthesis or signaling, calcium signaling, starch and sucrose synthesis and several other metabolic processes.

The studies on cassava identified 51 drought-specific differentially expressed lncRNAs and qRT-PCR validation of selected molecules among them revealed the up- regulation of lincRNA101, lincRNA391 and lincRNA356. Other lncRNAs, such as lincRNA64, lincRNA350, lincRNA182 and lincRNA392, were downregulated under drought stress. The TCONS_00060863 and TCONS_00097416 lncRNAs were shown to regulate ABA and ethylene signaling pathways, respectively, under drought stress [116]. In switchgrass, drought stress upregulated the lncRNAs XLOC_053020, XLOC_014465 and XLOC_033252 to control ABA synthesis, XLOC_074836 to regulate ethylene signaling and XLOC_005809 to control trehalose phosphate synthase [117].

In rice, 98 drought-responsive NAT-lncRNAs were identified by using RNA-Seq analysis. These included two important drought-responsive lncRNAs viz *NAT Os02g0250700-01* and *NAT Os02g0180800-01*, which targets the *late embryogenesis abundant protein* and *cinnamoyl CoA reductase* genes, respectively [119]. Studies in maize identified that the lncRNAs expressing at the R1 stage (silking stage) had a critical role in drought stress tolerance [122]. The possible role of lncRNAs as positive regulators of drought stress tolerance in *Arabidopsis* was identified with the discovery of a novel nucleus localized 755 nt long drought-induced lincRNA (*DRIR*). The *DRIR* overexpressing *Arabidopsis* lines had higher drought tolerance than wild-type seedlings [113]. This lincRNA was a nuclear-localized and controlled transcription of several drought stress-responsive genes, including ABA signaling genes (*ABI5*, *P5CS1*, *RD29A* and *RD29B*), aquaporin genes (*NIP1* and *TIP4*), annexin gene (*ANNAT7*), fucosyltransferase4 (*FUT4*) gene and transcription factor genes (*NAC3* and *WARKY8*) [113].

### 4.2. miRNAs in Drought Stress

Several studies have also shown the role of miRNAs in regulating plant response to drought stress (Table 4). A number of miRNAs, such as miR156, miR158, miR159, miR165, miR167, miR168, miR169, miR171, miR319, miR393, miR394 and miR396, were upregulated in response to drought stress in *Arabidopsis* [268]. The upregulation of *Arabidopsis* miR393, miR319 and miR397 in response to dehydration was reported earlier [224]. In drought-stressed rice seedlings, genome-wide analysis was carried out across different developmental stages, from tillering to inflorescence formation, using a microarray platform [269]. This analysis identified 30 miRNA gene families that were differentially regulated. Among these, 16 miRNA families, namely miR156, miR159, miR168, miR170, miR171, miR172, miR319, miR396, miR397, miR408, miR529, miR896, miR1030, miR1035, miR1050, miR1088 and miR1126, were significantly downregulated. Meanwhile, 14 miRNAs, namely miR159, miR169, miR171, miR319, miR395, miR474, miR845, miR851, miR854, miR896, miR901, miR903, miR1026 and miR1125, were significantly upregulated under drought stress. Another report showed that miR164c, miR319b and miR1861d were downregulated, while miR166h, miR172d and miR408 were upregulated under drought stress in rice [270]. In rice, pre-miRNA expression profiling indicated that miR171f was involved in the progression of rice root development and growth and response to drought stress [119]. In a different study, it was shown that miR169g was strongly upregulated and miR393 was transiently induced by drought stress in rice [271]. Inoculation of rice plants with *P. indica* caused upregulation of miR396, and this resulted in the downregulation of growth-regulating factor (GRF), which lowered the rate of transpiration and enabled the plants to tolerate drought [272].

In *Medicago truncatula*, miR169 was downregulated only in the roots while miR398a,b and miR408 were strongly upregulated in both shoots and roots under drought stress [299]. In a *Populus* plant, miR156, miR159, miR171, miR319, miR395 and miR474 were upregulated in response to drought stress [300]. In *Populus tomentosa*, about 152 conserved miRNAs were identified and the expression of 17 conserved and nine novel miRNAs was investigated in response to drought stress [301]. In *Vitis vinifera*, 12 novel and species-specific miRNA candidates were reported in response to drought stress. Moreover, 70 conserved miRNAs were identified and 28 novel miRNAs were predicted in a drought-resistant grapevine [302].

Differential regulation of miRNAs in response to drought stress has been well studied in maize [253,286,288,302,303,304]. For example, miR398 was upregulated after treatment with polyethylene glycol and downregulated under soil drought [305]. The downregulation of miR167 during drought stress upregulated its target PLD (Phospholipase D), which is involved in controlling ABA response and stomatal movement [21]. Similarly, the downregulation of miR159 in drought triggered the expression of HD-ZIP, ARF and GA-MYB transcription factors, which contributed to greater adventitious and lateral root formation. Moreover, miR474 was upregulated in drought to inhibit *proline dehydrogenase* (*PDH*) [306], while miR827 was upregulated during drought stress to act on *NADP-binding* and *SPX (SYG1/Pho81/XPR)* transcripts to activate stress signal transduction pathways [305].

It was shown that miR156 interacts with the ABA-dependent strigolactone signaling pathways in tomatoes under drought stress. The study identified miR156 as a mediator of stomatal movements and the findings indicated a cause–effect link between miR156 accumulation and regulation of water relations and stomatal functioning [307]. In sugarcane, miR169* was shown to target various transcripts such as *Elongation Factor 1-alpha* (*EF 1α*) in response to water depletion [297]. It was identified that miR529, miR535 and miR156 regulate transcripts of *Squamosa-promoter binding protein-like* (*SPL*) to control organ development and morphogenesis during stress. Similarly, miR159 targets *MYB33* and miR172 targets *AP2* (*Apetala 2*) to regulate plant development in response to drought stress [152].

Functional studies have provided an insight into the role of miRNAs in regulating the response to drought stress. In *Arabidopsis*, overexpression of ath-miR169a [308] and gma-169c [309], which targets the *Nuclear factor Y-A* (*NFY*-*A*) resulted in increased drought stress sensitivity. In contrast, similar studies in tomatoes have reported that plants overexpressing sly-miR169c show negative regulation of stomatal movement, reduced leaf water loss and transpiration rate, and improved drought tolerance [310]. Overexpression of Osa-miR393, which targets the auxin-responsive *OsTIR1* and *OsAFB2*, lowered the tolerance of rice plants to salt and drought stress [230]. In another example, Osa-miR319 overexpression in creeping bentgrass led to greater tolerance to salinity and drought, by decreasing the expression of its putative target genes: *AsPCF5*, *AsPCF6*, *AsPCF8*, *AsTCP14* and *AsNAC60* [283]. In *Populus ussuriensis*, overexpression of Pu-miR172d significantly decreased stomatal density by directly repressing the expression of *PuGTL1* and *PuSDD1*. This resulted in increased water use efficiency and drought tolerance by reducing net photosynthetic rate, stomatal conductance and rate of transpiration [311]. This study showed that Pu-miR172d-*PuGTL1*-*PuSDD1* module played an important role in stomatal differentiation and acted as a potential target for creating drought-tolerant plants. Similar studies in other plants showed that overexpression of miR156 in Alfalfa [312], miR408 in chickpea [282], Osa-miR319a in creeping bentgrass [283], miR169 in tomato [310] and miR159 in potato [313] resulted in enhanced drought stress tolerance. Recently, it was shown that miR535 overexpressing and *CRISPR*/*Cas9* knockout rice showed enhanced stress tolerance when tested in presence of sodium chloride, polyethylene glycol, abscisic acid and dehydration stresses parameters [314].

### 4.3. Interaction between Long and Small ncRNAs in Drought Stress

Studies on the mechanism of action of lncRNAs have revealed their complex interaction with the small ncRNAs. Together, they form complex regulatory hubs for controlling various drought responsive pathways at the transcription, post-transcription and epigenome levels. Studies in Cassava showed that 11 drought-specific differentially expressed lncRNAs acted as target mimics for miR156, miR164, miR169 and miR172 [116]. Under drought stress, lincRNA340 acts as a target mimic of miR169 to enhance the expression of its target gene *NFY* [115]. The lncRNA, TCONS_00068353 acted as a target mimic for miR156k and miR172c to control several abiotic stress-responsive genes [116].

Many plant TEs contain stress-responsive cis-acting elements and produce lncRNAs in response to specific stress [315], and many of these are possible sources of small ncRNAs that can regulate both TE and non-TE transcripts based on sequence complementarity. In maize, eight drought-responsive lncRNAs acted as precursors of miRNAs [102]. It was shown that TE-derived epigenetically activated siRNAs (easiRNAs) participated in transcriptional silencing. In rice, TE-siRNA815 could induce a de novo DNA methylation process via the RdDM pathway [316]. The stress-downregulated Osa-miR820 originates from CACTA-TE [317] and targets de novo *DNA methyltransferase* (*DRM2*) transcripts. Overexpression of Osa-miR820 enhanced salt tolerance in rice plants [318]. It was also shown that ZmNAC111 expression is repressed by miniature inverted-repeat transposable element (MITE) through RdDM and H3K9 dimethylation during drought tolerance [121]. Overexpression of the *ZmaNAC111* gene boosted drought tolerance in maize seedlings [319]. This phenomenon has unveiled functional crosstalk between small ncRNAs and the TEs, indicating that novel stress-responsive regulatory networks may be operative in plants [70,320].

## 5. Conclusions and Perspectives

The steadily increasing world population has challenged the agricultural sector to produce a substantial amount of crops. However, crop productivity all over the world is anticipating challenges by the ever-changing climate, variable weather conditions and environmental stresses. The limited availability of water and global warming has increased the incidence of drought, making it a major contributor to agricultural losses. To tackle this problem and produce enough food to feed the growing world population, it is important to generate crops that can survive underwater limiting conditions and can evade drought stress. This process can be aided by a thorough understanding of plant responses to water deficit and drought stress.

The exciting discovery of RNA-mediated gene silencing has highlighted the role of long and small ncRNAs in maintaining the homeostasis of gene expression. Advances in RNA-Seq analysis, computational analysis and functional genomic studies have enabled the discovery of several long and small ncRNAs and facilitated the understanding of their regulations. However, their functional characterization and annotation are limited to select plant species. Though the studies on ncRNAs are still in their infancy, their discovery has unraveled a novel mechanism of gene regulation. The small ncRNAs, such as miRNAs, regulate various aspects of plant biology, while the long ncRNAs have a role in regulating the miRNAs by acting as target mimics, sponges or decoys. The ncRNAs normally work in highly complex and intricately connected networks to regulate plant growth and development. The small ncRNAs belong to large families where specific members may be associated with a definite development stage or response.

In the last few years, substantial progress has been made in deciphering the mechanisms of ncRNAs. It has been shown that the small ncRNAs have the ability to move systemically within the plant’s vasculature or locally from one cell to another. This was demonstrated by micro-grafting miR399 overexpressing *Arabidopsis* shoots on wild-type roots. The chimeric plants accumulated very high levels of mature miR399 species in the wild-type roots, where the primary transcripts were virtually absent. The chimeric plants showed downregulation of *PHO2* in the wild-type roots and Pi accumulation in the shoots. This indicated a role for the miRNAs in long-distance signaling for maintaining nutrient balance [321] The miR399 could not only move through the phloem tissues, but the transported molecules retained their biological activity in the recipient tissues. In another report, both ath-miR399d and its star sequence were identified as the mobile elements. During phosphate starvation, translocation by miR827 and miR2111a between shoots and roots was also demonstrated [322]. The long-distance mobility of miRNA species reflects on their potential in root–shoot communications during stress responses [323,324]. The miRNA shuttles may be operative in response to drought stress, as well. Indications towards this come from studies on gma-miR172, which is induced under salt and drought treatments. The miR172 cleaves/inhibits the transcript encoding AP2/EREBP-type transcription factor (*SSAC1*) to relieve inhibition of thiamine biosynthesis gene (*THI1*) that encodes a positive regulator of salt stress tolerance [325].

There is no doubt that the ncRNAs play a crucial role in regulating plant growth and stress responses. Many important issues remain to be answered, such as how do the ncRNAs move from the cells where they are produced and move into the recipient cells? How are the ncRNAs transported, and in what way are they protected from nucleolytic degradation during movement? Are there specific proteins or chemical tags which help them in such transfers?

The information related to long ncRNAs is still emerging, and there is still a lot more to discover with respect to their functions and regulations. Dedicated and systematic efforts will be required to understand how the ncRNAs networks operate in different crop plants over spatiotemporal boundaries and identify their association with response to drought and related stresses. It will be a lot more exciting to understand if they have any role in influencing inter-organ communications and stress responses. In this context, genetic screens and transgenic approaches will aid in unraveling their novel functionalities and features. It is envisaged that such studies will open up opportunities for designing efficient strategies for development of stress-tolerant crops.

## Figures and Tables

**Table 1:** Functions of lncRNAs identified in various plants.

Plant	lncRNA Name	Pathway	Functional Annotation	References
*Arabidopsis*	*IPS1* lncRNA	Phosphate homeostasis	Target mimic for miR399, which regulates *PHO2*, a negative regulator of the phosphate transporters	[112]
	*Hidden Treasure 1* (*HID1*)	Photomorphogenesis	Promotes photomorphogenesis in continuous red light by directly repressing *Phytochrome Interacting Factor 3 (PIF3)*	[100]
	*ASCO*-lncRNA and NSR	Alternate splicing module	Recognizes alternatively spliced mRNA targets	[111]
	Drought Induced lncRNAs (*DRIR*)	Drought response	Positively regulates several drought responsive transcripts such as ABA-signal transducers (*P5CS1, RD29A,B* and *ABI5*); annexins (*ANNAT7*) and aquaporins (*TIP4*, NIP1)	[113]
*Brassica napus,* (Q2 and Qinyou8)	XLOC_042431 and XLOC_071559	Hormone signaling	Targets *BnaC06g05090D* gene to regulate ethylene metabolism, IAA, Cytokinins and ABA signaling	[114]
	XLOC_ 095305 and XLOC_100682		Targets *BnaA01g17750D* genes to regulate alpha trehalose phosphate synthase	
Cassava (TMS60444 and Ku50)	lincRNA340	Target mimicry	miR169 target mimicry, also targets *Nuclear Factor Y* (*NF-Y*)	[115]
	TCONS_00003360, TCONS_00015102,	Signal transduction	Calcium and ABA signaling	[116]
	TCONS_00149293		Ethylene metabolism	
	TCONS_00097416	Hormone signaling and target mimicry	Targets *CSLD5*, *ERL1* and *SPCH* genes to modulate ethylene signaling;	
	TCONS_00069665		Targets *LAX2, HDG11* and *SCR* genes; and regulates expression by targeting miR156	
	TCONS_00060863 TCONS_00068353		Targets *CYP707A1* gene and regulates in ABA catabolism	
	TCONS_00040721	MiRNA target	Targets *GRF1*, *HB51* and *DOX1*; regulates gene expression by targeting miR156, miR164, miR169 and miR172	
*Cleistogenes songorica*	MSTRG.25585.13	Metabolic pathway	Regulates sucrose metabolism	[97]
	MSTRG.42613.1		Regulates starch metabolism	
	MSTRG.43964.1, MSTRG.4400.2	Hormone signaling and target mimicry	Targets ABA pathway and related genes, regulates miR164, miR166, miR393 and miR397a,b and act as endogenous target mimic	
*Panicum virgatum* (Alamo)	XLOC_033252	Hormone signaling	Regulates ABA synthesis and signaling by targeting *Pavir.Eb01847* gene	[117]
*Populus trichocarpa* (Nisqually 1)	lincRNA20, lincRNA2752, lincRNA2962, lincRNA1039, lincRNA3241	miRNA regulation	Control drought stress by regulating ptc-miR476 and ptc-miR169	[118]
*Oryza sativa* (DXWR)	lncRNAMSTRG69391	Transcription regulation	Regulates biological process by targeting genes encoding calmodulin	[96]
	lncRNA MSTRG41712 and MSTRG68635	Translation inhibition	Targeting genes encoding heat shock protein and mitochondrial carrier proteins	
	lncRNA MSTRG65848, MSTRG27834 and MSTRG46301	Differential regulation	Upregulated and downregulated the lncRNAs themselves; response to drought stress and targets several genes	
*Oryza sativa* cv (Ilmi)	NATOs02g0250700–01 andOs02g0180800–01		Regulate response to drought by targeting *Os02g0250600-01* (encodes highly abundant protein during late embryogenesis) and *Os02g0180700-01* (encodes Cinnamoyl-CoA reductase)	[119]
*Triticum aestivum*(Kiziltan and TR39477)	c70772_g2_i1 and c90557_g1_i1	lncRNA-miRNA-mRNA network	Targets *c69036_g1_i1* and *c9653_g1_i2* genes to regulate drought stress	[120]
*Zea mays*	Li_TCONS_00080887, Zhang_TCONS_00012690, Zhang_TCONS_00012690 625-646, Boerner_Z27kG1_14953, Boerner_Z27kG1_09751, Boerner_Z27kG1_15115, Boerner_Z27kG1_08283, Boerner_Z27kG1_16361, Boerner_Z27kG1_23317, Boerner_Z27kG1_13892, Boerner_Z27kG1_01046, Boerner_Z27kG1_22106, Boerner_Z27kG1_03819, Boerner_Z27kG1_17085, Boerner_Z27kG1_06707, Boerner_Z27kG1_17308, Boerner_Z27kG1_01291, Boerner_Z27kG1_22188, Boerner_Z27kG1_15675, Boerner_Z27kG1_06005, Zhang_TCONS_00011169, etc.	miRNA targets or decoys	Targets or decoys of zma-miR156e-3p, zma-miR156h-3p, zma-miR159c,d-3p, zma-miR159e-5p, zma-miR159e-5p, zma-miR160b,g-3p, zma-miR160c-3p, zma-miR160c-3p, zma-miR162-5p, zma-miR164b-3p, zma-miR164d-3p, zma-miR164e-3p, zma-miR166h-5p, zma-miR166i-5p, zma-miR166i-5p, zma-miR166n-5p, zma-miR169c-3p, zma-miR169f-3p, zma-miR169l-3p, zma-miR169m-3p, respectively, etc.	[59]
	Miniature inverted-repeat transposable element (MITE-ZmNAC111)	RNA-directed DNA methylation	Represses *ZmNAC111* expression and enhances drought tolerance	[121]
	lncRNAMSTRG6838.1	Transcription regulation	Targets *V-ATPase-* and *VPP4-*encoding genes and regulates transcription	[122]
	Zm*PHO2*, *PILNCR*1	Phosphate homeostasis	Targets of Zma-miR399 in response to low phosphate	[123]

**Table 3:** Some drought-responsive lncRNAs reported in different plants.

Plant	Number of Putative lncRNAs Identified	Platform of Identification	Functional Annotation	References
*Arabidopsis thaliana*	303	qRT-PCR	Responsive to heat, cold, drought and salt stress	[94]
	*Hidden Treasure 1* (HID1)	Northern blotting	Promote photomorphogenesis in continuous red light by directly repressing PIF3	[100]
	13,230	Transcriptome Analysis, published tiling array datasets	Response to drought, cold, high-salt and/or ABA treatments	[98]
*Banana*	8471	Transcriptome Analysis, HiSeq	Drought stress-response	[260]
Cassava	682	HiSeq 2500, qRT-PCR, CNCI, CPC	Hormone signal transduction, sucrose metabolism pathway, etc.	[115]
	124	qRT-PCR	Melatonin responsive, drought stress regulation, cellular metabolism, Calvin cycle, hormone regulation, etc.	[116]
	1379	qRT-PCR	Different roles	[261]
	56,840	RNA-Seq Transcriptome Analysis	Differential expression in cold or drought conditions	[262]
Chickpea	3457	RT-qPCR, PLncPRO	Differentially expressed under drought stress	[257]
*Cleistogenes songorica*	3397	HiSeq2500, CPC, CNCI, CPATqRT-PCR	Regulate drought stress response	[97]
*Dimocarpus longan Lour*	7643	Real-time qPCR	Early somatic embryogenesis	[134]
*Oryza sativa*	98	HiSeq 2500, qRT- PCR	Regulatory role in drought response	[119]
	3714	RT-qPCR, PLncPRO	Differentially expressed under drought stress	[242]
*Panicum virgatum* L	16,551	HiSeq2500, qRT-PCR	Regulate drought stress response	[117]
*Populus trichocarpa*	504	HiSeq™ 2000, RT-qPCR	Drought- stress response, putative targets and target mimics of miRNAs	[118]
*Pyrus betulifolia*	251	HiSeq 4000, CNCI, CPC, qRT-PCR	Regulate various metabolic processes	[263]
*Setaria italica*	19	HiSeq 2000, qRT- PCR	Control drought stress response	[259]
*Solanum lycopersicum*	521	RT-qPCR	Variety of biological processes via lncRNA-mRNA co-expression	[264]
*Triticum aestivum* (Kiziltan, TR39477 and TTD-22 varieties)	59,110, 57,944 and 40,858	HiSeq 2000, qRT- PCR	Differential expression under drought stress response in cultivated and wild varieties	[120]
*Zea mays*	1724	RT-qPCR	Regulatory role in drought response	[102]
	637	Ribosomal RNA depletion and ultra-deep total RNA sequencing	Regulatory roles in response to N stress	[265]
	1535	HiSeq 2500, qRT- PCR	Oxidoreductase activity, water binding and electron carrier activity	[122]
	1199	RiboMinus RNA-Seq	Control drought and salt stress	[266]
	1769	Strand-specific RNA sequencing,	NATs in drought stress response	[267]

**Table 4:** Drought-responsive miRNAs reported in different crop plants.

Plant Name	miRNAs	Target	Target Description	References
*Arabidopsis*	miR160		ARF	[273]
	miR165/166		HD-ZIPIII, CLP-1, RDD1, ABA signaling	[274,275,276]
	miR167		IAR3	[277]
	miR169		NFY-A, HAP2	[278]
	miR408		LAC	[279]
Barley	miR397a	*MLOC_54246.3*	LAC-23	[280]
	miR399	*MLOC_52822.6*	Phosphatase 2	
	Novel-m0406-3p	*MLOC_70587.1*	PHD finger protein	
		*LOC_50162.1*	Sucrose synthase 1	
		*MLOC_67419.2*	PBS1, Ser/Thr-protein kinas,	
		*MLOC_67450.11*	D27, beta-carotene isomerase	
		*MLOC_73965.1*	Homocysteine S methyltransferase 3	
	Novel-m0598-3p	*MLOC_34795.2*	RNA polymerase (25-kDa subunit)	
	Novel-m0624-3p	*MLOC_55820.2*	Pectinesterase	
	Novel-m0793-3p	*MLOC_52822.6*	Phosphatase 2	
	Novel-m1587-5p	*MLOC_56261.3*	ABC transporter C family member 2-like	
	Novel-m1738-3p	*MLOC_3895.3*	Dro1 (coding for early auxin response protein)	
	Novel-m1900-5p	*MLOC_16998.3*	Glycine-rich RNA-binding protein 10	
	Novel-m2311-5p	*MLOC_61629.2*	Transcription elongation factor, SPT6	
	Novel-m2328-3p	*MLOC_6972.2*	DNA crosslink repair 1A protein	
Chickpea	miR159		GA-MYB-like	[281]
	miR160		ARF 16 (Seed germination and post germination stages)	
	miR166		ATHB-15 (axillary meristem initiation, leaf and vascular development)	
	miR167		ABI 5 (Gynoecium and stamen development)	
	miR169		NFY-A (plant development and flowering timing; response to different biotic stresses)	
	miR171		NSP2 (response to abiotic stresses and floral development)	
	miR172		RAP2-7 (flowering time, floral organ identity and cold stress response)	
	miR393		AFB2 (susceptibility to virulent bacteria)	
	miR396		CP29 (leaf and cotyledon development)	
	miR408		Plantacyanin (regulation of DREB and other drought responsive gene)	[282]
Creeping bentgrass	miR319		TCP	[283]
*Cucumis sativus*	miR159	*T159*	MYB protein 306-like	[284]
	miR167	*T167*	ARF 8-like	
	miR170	*T170*	GRAS transcription factor	
	miR172	*T172*	Floral homeotic protein, APETALA 2-like	
	miR319	*T319*	Transcription factor, MYB75-like	
	b-miR-n-07	*TB7*	ATPase	
	b-miR-n10	*TB10*	GRAS transcription factor	
	b-miR-n24	*TB24*	DELLA protein GAI1-like	
	miR169	*T169*	NFY-A-1-like	
	miR395	*T395*	ATP sulfurylase 1	
	miR398	*T398*	Superoxide dismutase	
	csa-miR-n19	*TC19*	Pleiotropic drug resistance protein 2-like	
	miR168	*T168*	Argonaute 1A-like	
	miR396	*T396*	Endoribonuclease dicer homolog 1-like	
	b-miR-n02	*TB2*	Pre-mRNA-processing factor 17-like	
	b-miR-n20	*TB20*	Dicer-like protein 4-like	
*Euphrates poplar*	miR30a,b	*eugene3.00010640*	Electron carrier activity	[285]
	miR71*	*eugene3.00010640*	Electron carrier activity	
		*grail3.0008024501*	Electron carrier activity	
		*eugene3.105640001*	Electron carrier activity	
		*fgenesh4_pg.C_scaffold_263000013*	Electron carrier activity	
	miR77	*eugene3.00002056*	Electron carrier activity	
		*estExt_Genewise1_v1.C_LG_XIV3469*	Electron carrier activity	
	miR84*	*fgenesh4_pm.C_LG_XIII000061*	Electron carrier activity	
	miR101a	*gw1.I.9350.1*	Transcription factor	
	miR131	*eugene3.00120942*	Electron carrier activity	
		*fgenesh4_pg.C_LG_X001404*	DNA binding	
		*estExt_Genewise1_v1.C_LG_XV2187*	Electron carrier activity	
		*fgenesh4_pg.C_scaffold_9189000001*	Electron carrier activity	
		*fgenesh4_pg.C_LG_II001303*	DNA binding	
	miR58	*estExt_Genewise1_v1.C_LG_XV2187*	SBP-box Transcription factor	
	miR67*	*gw1.VIII.1137.1*	Function unknown	
		*eugene3.00031501*	Vesicle transport v-SNARE	
	miR93a	*grail3.0010018301*	Function unknown	
		*estExt_Genewise1_v1.C_LG_IV3721*	NADH-ubiquinone oxidoreductase	
	miR93b	*grail3.0010018301*	Function unknown	
	miR106*	*estExt_fgenesh4_pg.C_17020003*	Cytochrome oxidase biogenesis protein	
		*estExt_fgenesh4_pm.C_1230037*	Function unknown	
	miR115a	*gw1.57.264.1*	Function unknown	
	miR123a	*estExt_fgenesh4_pg.C_LG_III1182*	Development and cell-death domain	
Maize	miR156c		Putative protein phosphatase 2C	[286]
	miR159a,b		Serine/threonine protein phosphatase	
	miR159a-d		GA-MYB transcription factor	
	miR160a-e		S16, 40S ribosomal protein	
	miR160b,i		ARR11, response regulator	
	miR166l,m		Homeodomain–leucine zipper protein	
	miR167a-i		ARF 12	
	miR167c		ARF 17, Putative eIF3e	
	miR167f,g		ARF 25	
	miR167d		Phospholipase D	
	miR168a,b		Serine/threonine-protein phosphatase	
	miR168b		Receptor-like protein kinase	
	miR168a,b		AGO1-1, mitogen-activated protein kinase 13	
	miR396a,b	*TC250636*	DEAD-box ATP-dependent RNA helicase 3,	
		*TC251979*	Putative early responsive to dehydration stress protein,	
		*TC274109*	GTPase,	
		*TC259098*	Heat shock protein 90,	
		*TC26999*	GA-MYB-binding protein	
	miR396d,e		Putative serine/threonine protein kinase	
	miR398b	*TC248005*	Pyruvate, orthophosphate dikinase,	
		*TC253981*	Putative protein serine/threonine kinase,	
		*TC270251*	Putative selenium binding protein,	
		*TC270802*	Fructose-bisphosphate aldolase	
	miR408		Leucine-rich repeat family protein	
	miR474b	*CF008935*	Putative CBL-interacting protein kinase,	
		*TC263244*	Proline dehydrogenase family protein,	
	miR474c	*CF055555*	Putative transcription factor MYB,	
		*CF632829*	WRKY transcription factor 31	
	miR528	*TC250873*	Cu/Zn SOD,	
		*TC274952*	Peroxidase	
	MiR827		N/Pi metabolism	
	miR156a/b,c,d,e,g,h,k,l	*AC233751, GRMZM2G061734, GRMZM2G065451*	DNA-binding putative protein	[159]
		*GRMZM2G040785),*	Unknown	
		*GRMZM2G307588*	SPL 6	
		*GRMZM2G414805*	SPL 11	
		*GRMZM2G460544*	SPL 7	
		*GRMZM2G067624*	Homoserine kinase	
		*GRMZM2G465165*	Serine/threonine protein kinase	
	miR159a,b,f,c	*GRMZM2G167088 and GRMZM2G416652*	DNA-binding protein	
		*GRMZM2G027100*	Unknown	
		*AC217264*	MYB55	
	miR159a,b,f and miR319a,c	*GRMZM2G028054*	GA-MYB	
	miR159a,b,f	*GRMZM2G423833, GRMZM2G075064*	DNA-binding protein	
	miR166d	*AC187157*	MPPN domain	
		*GRMZM2G003509*	Protein methyltransferase	
		*GRMZM2G499154*	Metabolic process	
	miR167a,c	*GRMZM2G078274, GRMZM2G475882*	Hormone stimulus	
	miR395b	*GRMZM2G04217*	Secondary active sulfate transmembrane transporter (1)	
		*GRMZM2G149952, GRMZM2G051270*	ATP sulfurylase	
	miR396f	*GRMZM2G178990*	Actin binding protein	
	miR1432	*GRMZM2G423139*	Calcium-binding allergen Ole e 8	
	miR1436	*GRMZM2G125531*	RNA binding protein	
	miR2097-5p	*GRMZM2G151955*	Serine/threonine protein kinase	
	mir319a-d-3p	*GRMZM2G089361TOl*	TCP family transcription factor	[287]
		*GRMZM2G145112 T02, GRMZM2G100579 T02*	Putative uncharacterized protein	
	miR393ac-5p	*GRMZM2G135978 Tol, GRMZM5G848945_T02*	Transport inhibitor response 1-like protein	
	miR396cd	*GRMZM2G033612 T02*	Putative uncharacterized protein	
		*GRMZM2G098594_ T06*, *GRMZM2G099862_ T04*, *GRMZM2G119359_T01*, *GRMZM5G893117 T01*, *GRMZM2G105335_ T02*, *GRMZM2G067743_T03*	GRF-transcription factor	
		*GRMZM2G029323_T01*	AP2/EREBP transcription factor protein	
	miR398ab-3p	*GRMZM2G023847 Tol, GRMZM2G097851 Tol*	Putative uncharacterized protein	
		*GRMZM2G352678 T01*	Chemocyanin	
		*GRMZM5G866053_T01*	Basic blue protein-like	
		*GRMZM2G122302_T01*, *GRMZM2G082940_T01*	Blue copper protein	
	miR444ab	*GRMZM2G492156_T01*, *GRMZM2G033093_T01*	MADS-box transcription factor	
		*GRMZM2G005000 T02*	Putative uncharacterized protein	
	miR168a-3p	*GRMZM2G369839 To1*	Putative uncharacterized protein	
	miR168b-3p	*GRMZM2G136486 T02*	Putative uncharacterized protein	
	miR319a-d-3p	*GRMZM2G020805_T01*	TCP family transcription factor	
	miR390ab-3p	*GRMZM2Gl07498_T01*	Putative uncharacterized protein	
	miR827-3p	*GRMZM2G175406_T01*	Putative uncharacterized protein	
	miR399	*PHO2* *, UBC24*	Control Pi homeostasis	[288]
	miR529		SPB domain transcription factor	
	miR399	*PHO2, UBC24*	Control Pi homeostasis	
	miR529		SPB domain transcription factor	
	miR156	*SPL*	Shoot development and delayed change in vegetative phase	[288,289]
	miR160		ARF (root development and auxin signals)	
	miR166		HD-ZIPIII (leaf development and polarity)	
	miR169	*HAP2*	Nitrogen homeostasis and stress response	
	miR395	*APS*, *AST*	Control ATP Sulfurylase activity	
	miR171	*SCL*	Regulate root development	[289]
	miR172	*AP2*	Maintain nitrogen remobilization and floral development	
	miR167		CCAAT-binding factor, ARF	
	miR397		LAC (regulate copper homeostasis and reduces root growth)	
	miR159	*MYB*	Regulate flowering time; leaf shape and size	[288]
	miR162	*DCL1*	Negative feedback regulatory function	[258]
	miR164	*NAC1*	Control lateral root development	[258,288]
	miR168	*AGO1*	Nutrient homeostasis and feedback regulation	[290]
	miR2275	*gnl|GNOMON|55702013.m*	Mitochondrial protein	[254]
	miR393	*gnl|GNOMON|39086093.m*	Protein transport inhibitor response 1-like	
	miR398	*CSD*	Copper homeostasis and oxidative stress	[291]
	miR156k		↓ in drought and submergence	[292]
	miR159ab		↑ in drought, ↓ in submergence	
	miR164e		↓ in drought and submergence	
	miR166b,d		↓ in drought and submergence	
	miR167c,d,e,g		↓ in drought and submergence	
	miR169c,r		↓ in drought and submergence	
	miR319b		↑ in drought, ↓ in submergence	
	miR396c,d		↓ in drought and submergence	
	miR398a,b		↓ in drought and submergence	
	miR398b		↓ in drought and submergence	
	miR408		↓ in drought and submergence	
	miR408b		↓ in drought and submergence	
	miR528ab		↓ in drought and submergence	
	miR166c		Constitutive expression	
*Medicago sativa*	miR156		SBP-like protein	[293]
*Medicago truncatula*	miR164		NAC domain transcription factor (lateral root development) ↓	[294]
	miR169		CBF (response to drought, cold and salinity, nodule development) ↓	
	miR171		GRAS transcription factors (response to drought, cold and salinity, nodule Morphogenesis and floral development) ↓	
	miR396		GRF (response to drought and salt; cell proliferation) ↓	
	miR398		Cu/Zn CSD1, CSD2 (response to oxidative stress) ↓	
	miR399	*PHO2*	ubiquitin conjugating enzyme balance of phosphorus, ↑	
	miR2118		TIR–NBS–LRR domain protein encoding response to drought, cold, salinity and ABA, ↑	
	miR1510a		PDC isozyme 1, concanavalin A-like lectin/glucanase 3. F-box protein, ↓	
	miR2089		NB–ARC domain protein, ↑	
	miR2111a-s,u-v		Calcineurin-like phosphoesterase, membrane protein SAK, ↑	
	miR5274b		DNA-damage-repair, toleration protein, ↑	
	miR5554a- c		Polynucleotidyl transferase, ribonuclease H fold, ↓	
	miR5558		Initiation factor eIF-4 gamma, homeodomain-related POX, ↑	
Rice	66 miRNAs		Response to drought stress	[119]
	miR167, miR9774, miR398, miR162, miR319, miR156, miR408, miR166, miR531, miR827 and miR8175		↓ expression profiling in response to drought stress	[294]
	miR6300, miR160, miR1861, miR440, miR9773, miR3982, miR171 and miR1876		↑ expression profiling in response to drought stress	
	67 novel drought responsive miRNAs		27 novel miRNAs ↓ and 40 novel miRNAs ↑ in response to drought stress	[295]
	Osa-miR159f, Osa-miR1871, Osa-miR398b, Osa-miR408-3p, Osa-miR2878-5p, Osa-miR528-5p and Osa-miR397a		↑ in the flag-leaves of tolerant cultivar (N22 and Vandana, while ↓ in sensitive cultivar (PB1 and IR64) during drought	[296]
	miR398	*CSD*	Regulate copper homeostasis and oxidative stress	[292]
Sugarcane	MiR160, miR399 and miR528		↑ in tolerant cultivar (RB867515)	[297]
	miR160, miR394, miR399 and miR1432		↑ in sensitive cultivar (RB855536)	
	miR166, miR169, miR171, MiR172, miR393, miR396, miR399 and miR1432		↓ in tolerant cultivar (RB867515)	
	miR166, miR171, miR396		↓ in sensitive cultivar (RB855536)	
Sunflower	miR399a-2	*HannXRQ_chr02g0057111*	Environment adaptation; leaf ↑; root ↑	[251]
	Novel-mir40 4	*HannXRQ_chr03g0090941*	DNA repair protein XRCC; root ↑	
	Novel-mir3, Novel-mir42	*HannXRQ_chr04g0098561*	Putative toll/interleukin-1 receptor; root ↑	
	miR396b	*HannXRQ_chr04g0115781*	Serine/threonine protein kinase; leaf ↑	
	miR156a-5p,f,k,q, 157a-5p	*HannXRQ_chr05g0138971*	SBP transcription factor; leaf ↑	
	Novel-mir3	*HannXRQ_chr05g0149501*	P-loop containing nucleoside triphosphate hydrolase; leaf ↓	
	miR396a,b-5p	*HannXRQ_chr05g0150421*	Glutamyl tRNA reductase and chlorophyll metabolism; leaf ↓	
	miR156h	*HannXRQ_chr07g0196531*	Leaf ↓	
	miR396f-1	*HannXRQ_chr08g0211484*	Serine/threonine dual specificity protein kinase; root ↑	
	miR394a-3p-1	*HannXRQ_chr08g0216701*	Related to Zn ion transport; leaf ↑	
	Novel-mir36	*HannXRQ_chr08g0219981*	Putative plant disease resistance response protein; root ↓	
	Novel-mir42	*HannXRQ_chr09g0239281*	Putative toll/interleukin-1 receptor homology (TIR) domain; root ↑	
	Novel-mir3	*HannXRQ_chr09g0239531*	P-loop containing nucleoside triphosphate hydrolase; root ↑	
	Novel-mir55	*HannXRQ_chr09g0252001*	C-terminal LisH motif-containing protein, Leaf ↑; root ↑	
	Novel-mir42	*HannXRQ_chr13g0396521*	P-loop containing nucleoside triphosphate hydrolase; root ↑	
	Novel-mir3	*HannXRQ_chr13g0396531*	Putative toll/interleukin-1 receptor; leaf ↓	
	Novel-mir65	*HannXRQ_chr14g0435381*	Root ↑	
	Novel-mir66	*HannXRQ_chr14g0435571*	Auxin-induced protein, leaf ↑	
	MiR172a-2	*HannXRQ_chr15g0491641*	Leaf ↑; root ↑	
	MiR156a-2	*HannXRQ_chr17g0534011*	(S)-urea glycine amidohydrolase; leaf ↑	
	Novel-mir17	*HannXRQ_chr17g0569261*	Probable response regulator 11; root ↓	
*Triticum aestivum*	miR156		SPL; leaf ↑; root ↑	[298]
	miR159		MYB transcription factor, leaf ↑; root ↓	
	miR160		ARF, leaf ↑; root ↑	
	miR162		GTPase activating protein-like; leaf ↑	
	miR164		NAC domain-containing protein; leaf ↑; root ↑	
	miR169		CCAAT-box-transcription factor; leaf ↓; root ↑	
	miR172		APETALA2 transcription factor; leaf ↓; root ↓	
	miR319		MYB transcription factor; leaf ↑; root ↓	
	miR396		Heat shock protein; leaf ↓; root ↓	
	miR398		Cu/Zn superoxide dismutase; leaf ↑; root ↓	
	miR482		TPGR; leaf ↑; root ↑	
	miR528		Glyceraldehyde-3-phosphate dehydrogenase; leaf ↑; root ↓	
	miR838		Small heat shock protein (Mds1); leaf ↓	
	miR1120		Glyceraldehyde-3-phosphate dehydrogenase; leaf ↑	
	miR1169		Small GTP-binding protein; root ↑	
	miR1436		Glutathione S-transferase; root ↑	
	miR1450		Manganese superoxide dismutase; leaf ↓	
	miR2102		Calmodulin-binding family protein; root ↑	
	miR4393		ARF; leaf ↑; root ↓	
	miR4993		SKP1/ASK1-like protein; root ↑	
	miR5048		RPG1, serine/threonine protein kinase; root ↓	
	miR5049		Wpk4 protein kinase, leaf ↑; root ↑	
	miR5059		Heat shock protein; root ↑	
	miR5075		Serine/threonine protein kinase 3; root ↑	
	miR5083		Hydroxymethylglutaryl-CoA synthase; leaf ↑	
	miR5174		NBS–LRR genes, leaf ↑; root ↑	
	miR5175		Methylene-tetrahydrofolate reductase; leaf ↑	
	miR5205		Malate dehydrogenase, CBS domain-containing protein; leaf ↑	
	miR5568		Pathogenesis-related protein, leaf ↑; root ↓	
	miR6108		Glycosyltransferase; leaf ↑	
	37 miRNAs including 5 novel miRNAs		27 ↑, 10 ↓	[34]
*Zanthoxylum bungeanum*	miR396a-5p		Superoxide dismutase [Mn] 1, mitochondrial	[14]
	miR834		Superoxide dismutase [Fe], chloroplastic-like isoform X2	
	miR167a-3p		Peroxiredoxin-2E, chloroplastic (POD)	
	miR169b-3p		Catalase isozyme 1(CAT)	
	miR447a-3p		L-ascorbate peroxidase 3	
	miR773b-3p		Phospholipid hydroperoxide glutathione peroxidase 1, chloroplastic	
	miR397b		Delta-1-pyrroline-5-carboxylate synthase, key enzyme for the synthesis of proline	
	miR397b	*JAR1*	Jasmonic acid-amido synthetase (participate in the synthesis of jasmonic acid)	
	miR859		ABSCISIC ACID–INSENSITIVE 5-like protein 5, (regulate a variety of ABA responses, such as stomatal closure, plasma membrane permeability and water permeability)	
	miR5632-5p		Mitogen-activated protein kinase 1	
	miR1888a		Protein disulfide-isomerase 5-2 isoform X1	
	miR5638a		Respiratory burst oxidase homolog protein C (Citrus sinensis)	
	miR398a-3p		Probable nucleoredoxin 1	
	miR3434-3p		Translationally controlled tumor protein homolog; involved in the regulation of abscisic acid–mediated and calcium-mediated stomatal closure	

AP2, Apetala 2; ARF, auxin response factor; CBF, CCAAT Binding Factor; CBS, cystathionine beta synthase; CLP-1, Cysteine Protease-1; CSD, copper/zinc superoxide dismutase; DCL, dicer-like protein; GTP, guanosine triphosphate; HAP2, heme activator protein 2; HD-ZIPIII, Homeodomain Leucine Zipper III; IAR3, Indole-3-Acetic Acid-Ala Resistant 3; LAC, Laccases; LMW, low molecular weight; LRR, leucine-rich repeats; MYB, myeloblastosis; NBS–LRR, nucleoside binding site–leucine-rich repeat; NFY-A, Nuclear Transcription Factor Y Subunit Alpha; NLA, nitrogen limitation adaptation; PHD, plant homeodomain; PDC, pyruvate decarboxylase; RDD1, Rice D of Daily Fluctuations 1; SBP or SPL, Squamosa promoter binding protein-like; SCL, scarecrow-like; SKP1, S-phase kinase-associated protein 1; SOD, superoxide dismutase; TCP, Teosinte Branched/Cycloidea/Proliferating Cell Factors (PCF); TPGR, transmembrane proton gradient regulation; UBC24, ubiquitin-conjugating enzyme; E2, phosphate 2.
